# Supplementary material for: Peak expiratory flow rate and sarcopenia risk in older Indonesian people: A nationwide survey
Source: PLoS One. 2021 Feb 9;16(2):e0246179. doi: 10.1371/journal.pone.0246179 (PMC7872226; doi:10.1371/journal.pone.0246179)
Supplement: Attachment — Submitted filename: Response to Reviewers.docx [file pone.0246179.s001.docx]

Dr. Joerg Heber

Editor-in-Chief

*PLOS ONE*

Subject	: Editor Decision and Reviewer’s Comments

Paper ID	: PONE-D-20-24364

December 5^th^, 2020

Dear Dr. Joerg Heber,

Thank you very much for the review of our manuscript entitled “Peak expiratory flow rate and sarcopenia risk in older Indonesian people: a nationwide survey”.

We sincerely appreciate all valuable comments and suggestions. We have revised the manuscript to conform to the format of PLOS ONE style and have amended authors along with affiliation and contribution to the work.

We carefully responded to all reviewers’ comments point-by-point and have modified the manuscript accordingy. The revisions have been approved by all authors and it has been uploaded to the PLOS ONE editorial manager submission system labelled as ‘manuscript with track changes’ and ‘manuscript’. Grammatical errors, ambiguous sentences, or word redundancies have been corrected accordingly.

We look forward to your favourable response.

Sincerely,

Pei-Shan Tsai, PhD

Professor, College of Nursing,

Taipei Medical University

250 Wu Shing St., Taipei, 110 Taiwan

Responses to the reviewer’s comments:

1. Is the manuscript technically sound, and do the data support the conclusions? Reviewer #1: Yes; Reviewer #2: Yes.

Response:

I would like to thank the reviewer for the acknowledgement of this manuscript

2. Has the statistical analysis been performed appropriately and rigorously? Reviewer #1: Yes; Reviewer #2: Yes.

Response:

Thank you very much for the positive comments on statistical analysis performance.

3. Have the authors made all data underlying the findings in their manuscript fully available? Reviewer #1: Yes; Reviewer #2: No.

Response:

I would like to thank for the reviewer’s comments. I have presented the underlying data that was analyzed fully available.

4. Is the manuscript presented in an intelligible fashion and written in standard English? Reviewer #1: Yes; Reviewer #2: Yes.

Response:

Thank you very much for this constructive comments

Reviewer #1:

1. Title: Title seems to be suitable for the manuscript. Abstract: The abstract of the study is brief and understandable.

Response:

I appreciate this positive comment.

2. Introduction:
a. The purpose of the article and the aim was expressed in the right way. Albeit, please shorten some parts of the introduction to make it more straightforward. Also, reduce the references that you cited. You may wish to cite current guideline of European Working Group on Sarcopenia in Older People (EWGSOP) instead of reference 1. (Sarcopenia: revised European consensus on definition and diagnosis).

Response:

We have shortened the introduction to make it more concise by reorganising sentences and citations. Hence, the paragraph of introduction section is shortened into three paragraph from four. In addition, the number of references reduced to 47 from previously 52 references. We agreed to cite the guideline of The European Working Group on Sarcopenia in Older People (EWGSOP) by Cruz-Jentoft et al. (2010) as it was an original citation.

The revision highlighted as following:

a. Paragraph one, line 47 (reference 1).
b. Paragraph one, line 55 – 57.
c. Paragraph two, line 60.
d. Paragraph two, line 65 – 67.
e. Paragraph three, line 71 – 76.
3. Material and methods:
a. Add the date and number of ethical approval.
b. Authors mentioned that Gait speed was determined based on a timed walk on a 2.5-m straight flat surface. Please cite reference for this measurement. Do authors mean 4 or 6 meter walking speed?
c. What are the inclusion and exclusion criteria of the study?

Response:

a. Datasets that were analysed provided the ethical clearance number obtained from the Institutional Review Board of the Survey METER in Indonesia and Research and Development, Indonesia (RAND) for publication purposes. We presented the protocol approval number in study sample and procedure section, paragraph one, line 95 - 97.
b. Gait speed was based on the result of a timed walk of 2.5-m straight flat surface. Reference was cited and presented in the sarcopenia section, line 119.
c. Inclusion and exclusion criteria were added in study sample and procedure section, paragraph two, line 98 - 99.
4. Results:

What are the differences between sarcopenic and non-sarcopenic patients according to the PEFR <50%, 50% to 80%, and >80% of the predicted value? You had better write in table1.

Response:

The differences between sarcopenics and non-sarcopenics according to predicted PEFR <50%, 50% to 80%, and PEFR >80% are presented in table 1.

5. Discussion:
a. You may wish to cite some more recent papers, which support this manuscript’s findings (please see below-references section).
b. Line 237 and 238 ----- ‘We also demonstrated that handgrip strength was significantly associated with FEV1, maximal voluntary ventilation, and lung function (37, 38).’ Did you demonstrate that in your study?

Response:

a. We appreciate the reviewer’s valuable suggestion to cite recent papers supporting the finding.
b. We have clarified the statement in previously line 237 and 238 that stated ‘We also demonstrated that handgrip strength was significantly associated with FEV1, maximal voluntary ventilation, and lung function (37, 38).’ Our study did not demonstrate the measurement; it was a citation from the study of Zhu et al. (2019) and Son et al. (2018). The statement was revised to improve the quality of discussion.
6. References:
a. There is no reference from 2020. For an article that will be published this year, the authors may well wish to add some more recent papers (i.e. the very nice article on diaphragmatic muscle thickness and PEFR measurements in sarcopenic patients-Diaphragmatic muscle thickness in older people with and without sarcopenia, PMID: 32406014).
b. Try to reduce the references. Revise references, many of them are incorrect, including the abbreviations of the journals or non-sense words. See journal information for authors

Response:

a. We have cited a recent paper entitled ‘diaphragmatic muscle thickness in older people with and without sarcopenia, PMID: 32406014’. The paper supported our discussion in paragraph two, line 230 – 231.
b. Some incorrect abbreviations and non-sense words of reference lists were corrected to conform to PLOS ONE reference guideline.

Reviewer #2:

1. This is an observational study performed in a large sample of community-dwelling Indonesian individuals aged ≥60 years (n = 2422) aimed to analyses prevalence of sarcopenia and its relationship with peak flow rate as a useful biomarker for analysis of sarcopenia. There are some major concerns that need to be addressed in order to support the methodology and thus, the authors' conclusions. Authors need to include and cite all study performed in ASIA about sarcopenia prevalence in older individual only one reference has been cited (page 3 line 61).

Response:

We added a prevalence meta-analysis of sarcopenia in Japanese community-dwelling older population to support previously included evidence, in introduction section, paragraph one, line 55 - 57.

2. Authors should clarify how sarcopenia criteria were used to diagnose sarcopenia because one individuals fulfilled one or two criteria out 3 what happens?, an algorithm of diagnosis of sarcopenia should be included.

Response:

In our study, sarcopenia was determined based on the diagnosis criteria presented by Asian Working Group for Sarcopenia which categorized sarcopenia into two indicator: sarcopenia and non-sarcopenia. In the method section, sarcopenia part, line 109 - 112 explained the criteria used to diagnose sarcopenia. Algorithm of diagnosis sarcopenia adopted from AWGS was added as Fig. 2.

3. Sensitivity, specificity and alpha Cronbach data should be stated for all questionnaire used in the study.

Response:

Cronbach’s alpha values for internal consistency the questionnaire of sleep quality and depressive symptoms were added in the manuscript, in confounders section, paragraph one, line 154 - 157, and line 160 - 162.

4. The criteria used to classify individuals as having "high", "moderate", and "low" physical activity are not explained in details, cut off values (minutes/per week) should be included and referenced properly as it is a crucial variable in this study and in general in the sarcopenia research field.

Response:

The classification of physical activity revised with detail cut of point based on IPAQ guidelines and it was presented in the confounder section, paragraph one, line 149 - 151.

5. Relevant study published on respiratory function and sarcopenia have not been cited, hence the discussion is quite poor:
a. Landi F, Salini S, Zazzara MB, Martone AM, Fabrizi S, Bianchi M, Tosato M, Picca A, Calvani R, Marzetti E. Relationship between pulmonary function and physical performance among community-living people: results from Look-up 7+ study. J Cachexia Sarcopenia Muscle. 2020 Feb;11(1):38-45.
b. Martínez-Arnau FM, Buigues C, Fonfría-Vivas R, Cauli O. Respiratory Muscle Strengths and Their Association with Lean Mass and Handgrip Strengths in Older Institutionalized Individuals. J Clin Med. 2020 Aug 24;9(9):2727.
c. Pacifico J, Geerlings MAJ, Reijnierse EM, Phassouliotis C, Lim WK, Maier AB. Prevalence of sarcopenia as a comorbid disease: A systematic review and meta-analysis. Exp Gerontol. 2020 Mar;131:110801.

Response:

These published articles were important for our study. We appreciate this suggestion. We have cited these articles to improve the quality of our discussion. These articles were cited in the paragraph 2, discussion section, line 226 – 227, line 234– 235, and in paragraph five, line 284 – 286.
